# Clinical Relevance of Estrogen Reactivity in the Breast Cancer Microenvironment

**DOI:** 10.3389/fonc.2022.865024

**Published:** 2022-05-23

**Authors:** Takashi Takeshita, Yoshihisa Tokumaru, Masanori Oshi, Rongrong Wu, Ankit Patel, Wanqing Tian, Yutaka Hatanaka, Kanako C. Hatanaka, Li Yan, Kazuaki Takabe

**Affiliations:** ^1^ Department of Breast Surgery, Hokkaido University Hospital, Sapporo, Japan; ^2^ Breast Surgery, Department of Surgical Oncology, Roswell Park Comprehensive Cancer Center, Buffalo, NY, United States; ^3^ Department of Biostatistics and Bioinformatics, Roswell Park Comprehensive Cancer Center, Buffalo, NY, United States; ^4^ Research Division of Companion Diagnostics, Hokkaido University Hospital, Sapporo, Japan; ^5^ Department of Surgery, University at Buffalo Jacobs School of Medicine and Biomedical Sciences, The State University of New York, Buffalo, NY, United States; ^6^ Department of Breast Surgery and Oncology, Tokyo Medical University, Tokyo, Japan; ^7^ Department of Surgery, Yokohama City University, Yokohama, Japan; ^8^ Department of Surgery, Niigata University Graduate School of Medical and Dental Sciences, Niigata, Japan; ^9^ Department of Breast Surgery, Fukushima Medical University, Fukushima, Japan

**Keywords:** breast cancer, estrogen reactivity, cancer genomics, tumor immune microenvironment, bioinformatics

## Abstract

**Purpose:**

Estrogen signals play an important role in the phenotype of estrogen receptor-positive breast cancer. However, comprehensive analyses of the effect of responsiveness to estrogen signals on the tumor microenvironment and survival in large cohorts of primary breast cancer patients have been lacking. We aimed to test the hypothesis that estrogen reactivity affects gene expression and immune cell infiltration profiles in the tumor microenvironment and survival.

**Methods:**

A total of 3,098 breast cancer cases were analyzed: 1,904 from the Molecular Taxonomy of Breast Cancer (METABRIC) cohort, 1,082 from The Cancer Genome Atlas (TCGA) cohort, and 112 from the Hokkaido University Hospital cohort. We divided the group into estrogen reactivity-high and estrogen reactivity-low groups utilizing the scores of ESTROGEN_RESPONSE_EARLY and ESTROGEN_RESPONSE_LATE in Gene Set Variation Analysis.

**Results:**

Breast cancer with high estrogen reactivity was related to Myc targets, metabolism-related signaling, cell stress response, TGF-beta signaling, androgen response, and MTORC1 signaling gene sets in the tumor microenvironment. Low estrogen reactivity was related to immune-related proteins, IL2-STAT5 signaling, IL6-JAK-STAT3 signaling, KRAS signaling, cell cycle-related gene sets, and EMT. In addition, breast cancer with high levels of estrogen reactivity had low immune cytolytic activity and low levels of immunostimulatory cells. It also had low levels of stimulatory and inhibitory factors of the cancer immunity cycle. Patients with high estrogen reactivity were also associated with a better prognosis.

**Conclusion:**

We demonstrated the relationship between estrogen reactivity and the profiles of immune cells and gene expression, as well as survival.

## Introduction

Breast cancer (BC) has different subtypes of which approximately 75% are sensitive to the female sex hormone, estrogen (1). These types of BCs, known as estrogen receptor-positive (ER+) BC, express receptors that bind to estrogen which promotes cell growth and cancer progression. Estradiol [17β-estra-1,3,5(10)-triene-3,17-diol] (E2) is a predominant form of estrogen, which is produced by theca cells and granulosa cells in the ovaries. Epidemiologic and experimental data implicate E2 as an important factor contributing to BC carcinogenesis (2). E2 signaling is primarily mediated by two isotypes of the receptor, ERα and ERβ, both of which are nuclear transcription factors that bind to their specific ligand and several estrogens. E2 *via* ERα signaling stimulates cell proliferation, while ERβ activation inhibits cell proliferation and promotes apoptosis. E2 signaling is also primarily mediated by a membrane-anchored receptor called G protein-coupled estrogen receptor 1, in which target gene transcription occurs through secondary messengers and several transcription factors (3–5).

The cellular response to estrogens, estrogen reactivity, plays an important role in the molecular and genomic pathways of the hormone-responsive BC phenotype by affecting the transcriptional modulation of various genes such as proliferation regulators, growth factors, cell cycle, and apoptosis modulators (5). Thus, it has been deeply studied to try to characterize the structure of the process, and many advancements have been made (5, 6). We have demonstrated that high expressions of estrogen-responsive genes were deeply involved in the late recurrence of BC patients (7). On the other hand, the physical behavior of the extracellular matrix, stromal cells, fibroblasts, adipocytes, immune cells, and macrophages is known to modulate tumor cells and the tumor microenvironment (TME) to promote cancer survival and progression (8). In addition, researchers have focused not only on cancer cells themselves but also on TMEs as targets for cancer therapy (8). Therefore, it is of interest to investigate estrogen reactivity in the TME as well as in breast tumors.

However, a comprehensive analysis showing the relationship between responsiveness to estrogen signals and TME components such as immune cells and gene expression profiles, and how they affect survival in large cohorts of primary BC patients, has been lacking. Gene Set Variation Analysis (GSVA) is an analysis to further explore the biological activity of a signaling pathway, which assesses the impact of specific pathways on the bioactivity of a set of genes of interest (9). Utilizing ESTROGEN_RESPONSE_EARLY and ESTROGEN_RESPONSE_LATE scores in GSVA, we can line up the patients in the order of how much estrogen reactivity is constantly upregulated.

We aimed to test the hypothesis that estrogen reactivity affects gene expression and immune cell infiltration profiles in the TME, and survival, retrospectively, utilizing collected data from the Molecular Taxonomy of Breast Cancer International Consortium (METABRIC) and The Cancer Genome Atlas (TCGA) BC cohorts and our own institutional data.

## Materials and Methods

### Data Acquisition From METABRIC and TCGA

We obtained clinical and genome data of 1,904 patients in the METABRIC cohort through cBioPortal (METABRIC Nature 2012 & Nat Commun 2016 dataset) (10) and (11 dataset) (11). We also obtained transcriptomic and clinical data of 1,082 female patients who had a histopathological diagnosis of BC in TCGA cohort (12) through cBioPortal (TCGA Pan-Cancer Atlas dataset) (10, 13) and (14 dataset) (14). In METABRIC and TCGA, given that the patient data are de-identified, and that it is in a public domain, it waived Institutional Review Board approval.

### Tissue Samples

Formalin-fixed paraffin-embedded (FFPE) tissues were obtained from 112 BC patients, who underwent breast surgery between 2005 and 2012 at Hokkaido University Hospital. We selected these 112 patients whose ER, progesterone receptor (PgR), lymph node metastasis status, and adjuvant therapy were all known and whose gene quality was guaranteed.

### Extraction of RNA and Preparation for Analysis by Expression Microarray

FFPE tissues were stored until the study was performed, and four sections of 10-µm thickness of FFPE specimens were obtained for RNA extraction. RNA extraction was performed using the RNeasy FFPE Kit according to the manufacturer’s protocol (Qiagen, Hilden, Germany). The extracted RNA was synthesized into cDNA using the Ovation FFPE WTA System (Affymetrix, Santa Clara, CA, USA) and the Encore Biotin Module (Affymetrix) kits according to the manufacturer’s instructions. cDNA (100 ng) was then used for gene expression analysis by DNA microarray using the Human Genome U133 Plus 2.0 Array according to the manufacturer’s instructions (Affymetrix).

### Estimation of Estrogen Reactivity

The GSVA score of the “Hallmark estrogen response early” and “Hallmark estrogen response late” gene sets in the MSigDB Hallmark collection (15) was used to measure the estrogen reactivity. The GSVA Bioconductor package (version 3.10) was used (16).

### Cluster Analysis

We choose hierarchical clustering using the Euclidean distance and Ward’s linkage due to its relative good performance (17). The R-function “hclust” was used for performing hierarchical clustering.

### Statistical Analyses of RNA Expression and Gene Set Enrichment Analysis

The analysis followed a two-step process as previously described (7, 18). We first calculated the fold changes of genes, corresponding to a high relative to low estrogen reactivity, which provided a list of t-scores and corresponding P-values for high relative to low estrogen reactivity in relation to each of the gene’s expression values. In the second step, gene set enrichment analysis (GSEA) was performed in GSEA pre-ranked using the collections of gene sets from the Hallmark gene sets using software provided by the Broad Institute (http://software.broadinstitute.org/gsea/index.jsp). We only considered significantly enriched gene sets that met a threshold of normalized enrichment score (NES) >1.8 or <-1.8 and false discovery rate (FDR) q-value < 0.01.

### Tumor Immune Microenvironment Analysis

The CIBERSORT deconvolution algorithm (19) was used to estimate the fraction of immune cells in TME using transcriptomic data of the whole tumor *via* an online calculator (https://cibersort.stanford.edu/), as previously shown (7, 18, 20). xCell (21), which is a bioinformatics tool that performs cell type enrichment analysis from gene expression data for 64 immune and stroma cell types, was used to support the predictions made by CIBRSORT using transcriptomic data of the whole tumor *via* an online calculator (https://cibersort.stanford.edu/). Immune cytolytic activity (CYT) was defined as the geometric mean of GZMA and PRF1 expression values in Transcripts Per Million (22, 23), and CYT was calculated as previously described (7, 18, 20, 24–28).

### Statistical Analysis

All statistical analyses were performed using R software (http:///www.r-project.org/) and Bioconductor (http://bioconductor.org/). The chi-square test or Fisher’s exact test or the non-parametric Mann–Whitney U test and contingency analysis were used to assess baseline differences between binary variables. Correlations were calculated using Spearman’s rank correlation coefficient. In the analysis of recurrence-free survival (RFS), the Kaplan–Meier method was used to estimate survival rates, and differences between survival curves were evaluated by the log-rank test. Two-sided p-values <0.05 were considered as statistically significant for all tests.

## Results

A total of 3,098 BC cases were analyzed: 1,904 from the METABRIC cohort, 1,082 from TCGA cohort, and 112 from the Hokkaido University Hospital cohort. We investigated the clinical relevance of estrogen reactivity in these BC cohorts based on the scores of ESTROGEN_RESPONSE_EARLY and ESTROGEN_RESPONSE_LATE in GSVA. The ESTROGEN_RESPONSE_EARLY gene set contained 200 genes which react early to estrogen, and the ESTROGEN_RESPONSE_LATE gene set contained 200 genes which react late to estrogen. The scores of ESTROGEN_RESPONSE_EARLY and ESTROGEN_RESPONSE_LATE were the highest in the hormone receptor (HR)+HER2- subgroup in METABRIC and TCGA (**Figure S1**). There was a strong correlation between ESTROGEN_RESPONSE_EARLY score and ESTROGEN_RESPONSE_LATE score in METABRIC and in TCGA, as well as in our institutional cohort (**Figure S2**).

**Figure 1:**
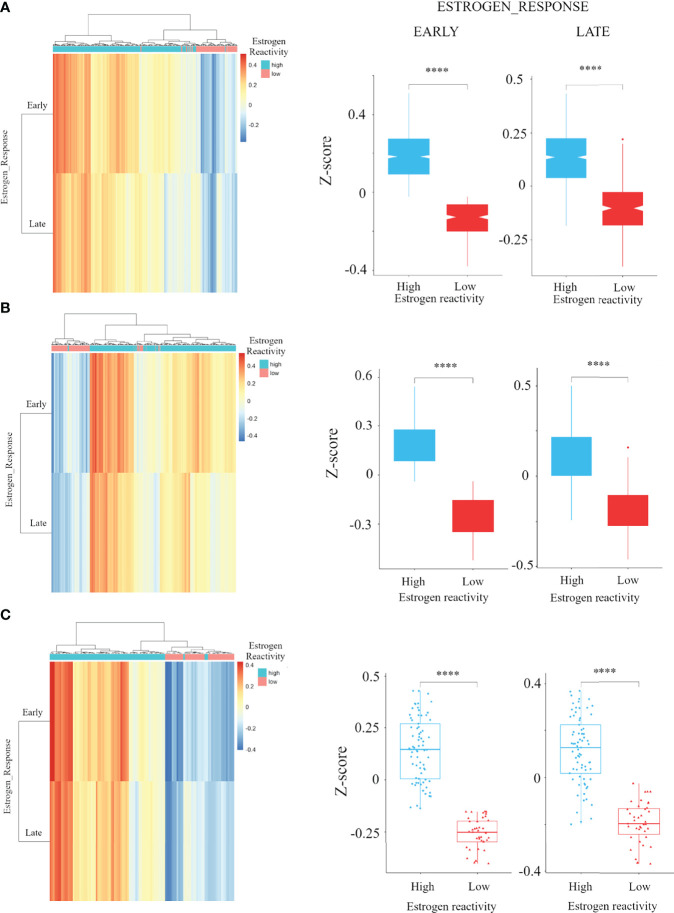
The association of estrogen reactivity and estrogen response scores in GSVA. Heatmaps and boxplots of the comparison of estrogen response early and late score by estrogen reactivity in the METABRIC cohort **(A)** TCGA cohorts **(B)** and our cohort **(C)** were shown. In heatmap, the scores of ESTROGEN_RESPONSE_EARLY and ESTROGEN_RESPONSE_LATE are displayed as colors ranging from red to blue as shown in the key. Both rows and columns are clustered using correlation distance and average linkage. **** means P < 0.0001. GSVA, Gene Set Variant Analysis; ER, estrogen receptor; PgR, progesterone receptor; METABRIC, Molecular Taxonomy of Breast Cancer International Consortium; TCGA, The Cancer Genome Atlas; HR, hormone receptor; HER2, human epidermal growth receptor 2; TN, triple negative.

We performed hierarchical clustering on ESTROGEN_RESPONSE_EARLY score and ESTROGEN_RESPONSE_LATE score for classifying the group into estrogen reactivity-high and estrogen reactivity-low groups in the HR+ subgroup, which was 1,016/1,355 (75%) in the HR+HER2- in METABRIC, 440/585 (75.2%) in the HR+HER2- in TCGA, and 73/112 (65.2%) in our cohort. This cutoff value could dichotomize the ESTROGEN_RESPONSE_EARLY score and ESTROGEN_RESPONSE_LATE score of BC patients based on statistical significance in the whole cohort and each among each subtype in METABRIC, TCGA, and in our cohort (**Figures 1** and **S3**). In an immunohistochemistry study for ER and PgR, there was no difference in percentage of ER due to estrogen reactivity, but a high estrogen reactivity was statistically significantly associated with a higher percentage of PgR, which is an estrogen-responsive gene (**Figure S4**). These results indicate that estrogen reactivity can be quantified utilizing ESTROGEN_RESPONSE_EARLY score and ESTROGEN_RESPONSE_LATE score in GSVA.

### Association of Estrogen Reactivity With Clinical Features

We studied the relationship between clinical features of the primary tumor and the levels of estrogen reactivity in the HR+ HER2- subgroup in METABRIC, TCGA (**Table S1**), and our cohort (**Table S2**). In METABRIC, patients with high estrogen reactivity were significantly associated with lower age (P = 0.002), premenopausal state (P = 0.002), PgR+ (P < 0.0001), and luminal A subtype (P < 0.0001). In TCGA, patients with high estrogen reactivity were significantly associated with lower age (P = 0.037), premenopausal state (P = 0.0075), and PgR positive (P < 0.0001). In our cohort, there was no significant differences in clinical features between patients with high and low estrogen reactivity. These results indicate that high estrogen reactivity was related to lower age, premenopausal state, and PgR positivity.

### Gene Expression Profiles by the Levels of Estrogen Reactivity

**Figure 2:**
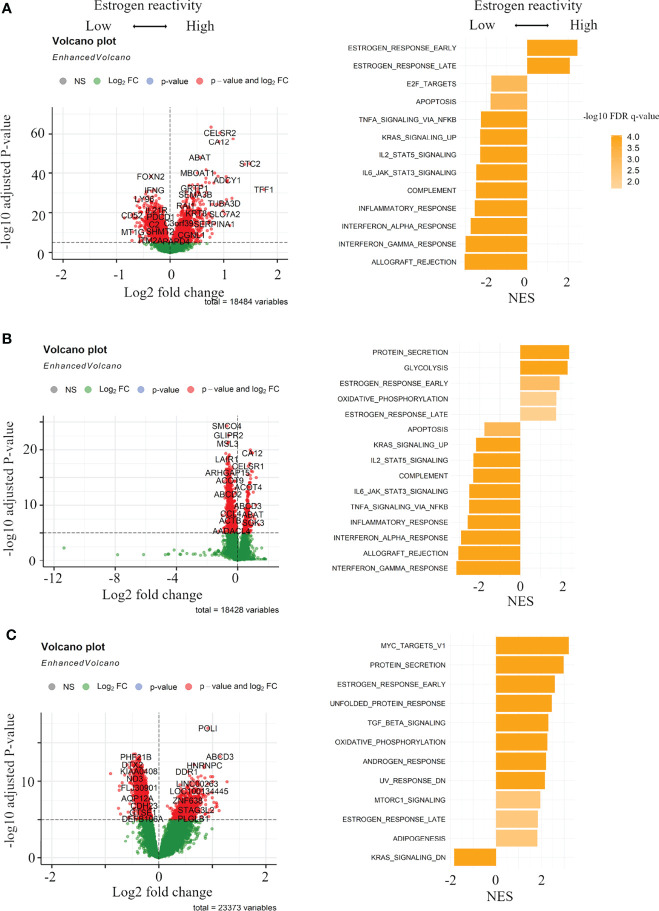
Gene expression profiles in the levels of estrogen reactivity. Volcano plots illustrating the differentially expressed mRNAs of BC and pre-ranked GSEA of BC patients comparing high vs. low estrogen reactivity in METABRIC cohort **(A)** and TCGA BC cohort **(B)** and our institutional cohort **(C)**. Left panels: In volcano plots, X-axes: log2 FC; Y-axes: -log 10 P-value from limma analysis. mRNAs with P-value < 0.05 in red, all others in green. Right panels: in pre-ranked GSEA, orange bar shows NES and its shading shows the –log10 FDR q-value. We only considered gene sets significantly enriched that met a threshold of NES >1.8 or <-1.8 and FDR q-value < 0.01. BC, breast cancer; GSEA, Gene Set Enrichment Analyses; METABRIC, Molecular Taxonomy of Breast Cancer International Consortium; TCGA, The Cancer Genome Atlas; FC, fold change; NES, normalized enrichment score; FDR, false discovery rate; HR, hormone receptor.

In order to clarify the mechanisms associated with the levels of estrogen reactivity, volcano plots and pre-ranked GSEA were performed in all cohorts. **Figure 2** shows volcano plots that represent the distribution of the fold changes and adjusted P-values of 18,484 genes in the METABRIC HR+HER2- cohort, 18,428 genes in TCGA HR+HER2- cohort, and 23,373 genes in our cohort corresponding to the levels of estrogen reactivity. In the high estrogen reactivity group, 3,747 mRNAs in the METABRIC HR+HER2- cohort were upregulated and 4,864 mRNAs were downregulated (**Figure 2A**). In TCGA HR+HER2- cohort, 2,020 mRNAs were upregulated and 6,020 mRNAs were downregulated (**Figure 2B**). A total of 4,504 mRNAs in our cohort were upregulated, and 9,900 mRNAs in our cohort were downregulated (**Figure 2C**), all of which were differentially expressed with P < 0.05.

The pre-ranked GSEA shows that, in the high estrogen-reactive group, in all cohorts, early and late estrogen responses were enriched (**Figures 2A–C**; **Tables S3** **-5**). Additionally, in our cohort, Myc targets v1, metabolism-related gene sets (protein secretion, oxidative phosphorylation, adipogenesis), cell stress-responsive gene sets (unfolded protein response, UV response down), TGF-beta signaling, androgen response, and MTORC1 signaling were enriched (**Figure 2C**; **Table S5**). In the low estrogen-responsive group, in METABRIC and TCGA, immune-related proteins (allograft rejection, interferon (IFN)-α/-γ response, inflammatory response, and complement), IL2-STAT5 signaling, IL6-JAK-STAT3 signaling, and KRAS signaling up were enriched (**Figures 2A, B**; **Tables S3**, **S4**). Additionally, in METABRIC, cell cycle-related gene sets (E2F targets, G2M checkpoint) and, in TCGA, epithelial–mesenchymal transition (EMT) and, in our cohort, KRAS signaling down were enriched. These results indicate that high estrogen reactivity was related to Myc targets, metabolism-related gene sets, TGF-beta signaling, androgen response, UV response down (stress-related pathway), and MTORC1 signaling. Low estrogen reactivity was related to immune-related proteins, IL2-STAT5 signaling, IL6-JAK-STAT3 signaling, KRAS signaling, cell cycle-related gene sets, and EMT.

### High Estrogen Reactivity Was Associated With Low Immune Cytolytic Activity and Low Levels of Immunostimulatory Cells

**Figure 3:**
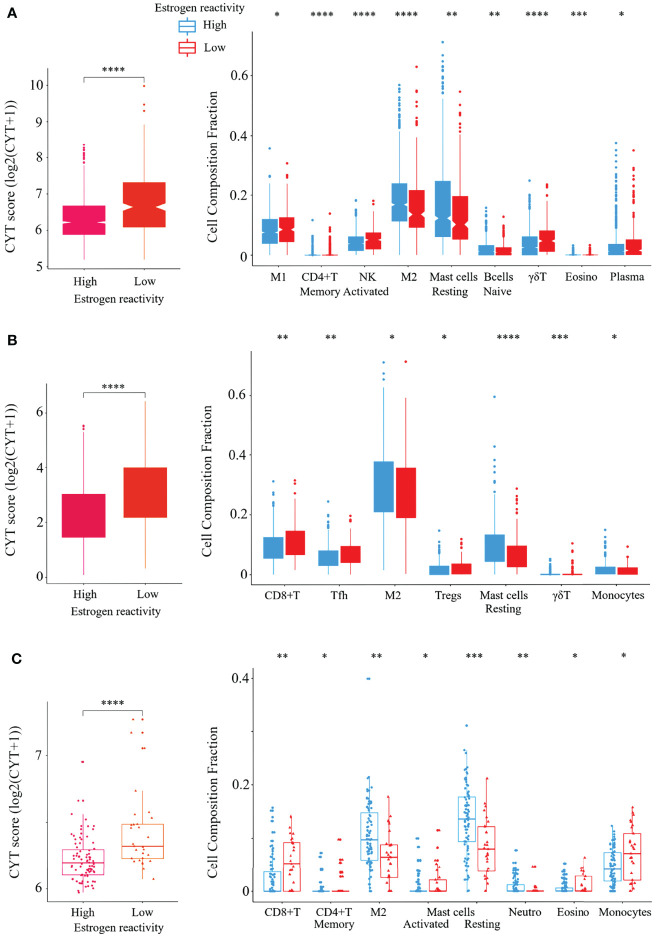
Verification of the relationship between estrogen reactivity and CYT and immune cell fractions. Box plots of the relationship between estrogen reactivity and CYT; left panel and immune cell fractions; right panel in METABRIC **(A)** and TCGA **(B)** and our cohort **(C)** were shown. **** means P < 0.0001, *** means P < 0.001, ** means P < 0.01, and * means P < 0.05. CYT, immune cytolytic activity; METABRIC, Molecular Taxonomy of Breast Cancer International Consortium; TCGA, The Cancer Genome Atlas; M1, M1 macrophage; NK, activated NK cells; M2, M2 macrophage; Tfh, follicular helper cells; Tregs, CD4+ regulatory T cells.

In order to investigate the tumor immune microenvironment by estrogen reactivity, we analyzed CYT and the immune cell composition utilizing CIBERSORT and xCell in all cohorts (**Figure 3**). It has been well established that the CYT score represents the overall cytolytic activity of immune effector cells in a bulk tumor (22). We found that CYT scores in BC tumors were significantly lower in the high estrogen reactivity group in all cohorts. Among the immunostimulatory cells, M1 macrophages and activated NK cells were lower within the high estrogen reactivity group in the METABRIC cohort. CD8+ T-cells were lower in both TCGA and our cohorts. Activated CD4+ memory T cells were lower in both METABRIC and our cohorts. Follicular helper T cells were lower in TCGA. In the analysis by xCell, CD8+ T-cells and M1 macrophages were lower within the high estrogen reactivity group in METABRIC and TCGA. CD4+ memory T cells were lower in all cohorts (). Among the immunosuppressive cells, M2 macrophages were higher within the high estrogen reactivity group in all cohorts. Regulatory T cells (Tregs) were lower in TCGA. In the analysis by xCell, M2 macrophages and Tregs were lower within the high estrogen reactivity group in METABRIC and not significant in TCGA and our cohort (). In terms of the other immune cells, resting MAST cells were lower within the high estrogen reactivity group in all cohorts. Γδ T cells were lower in both METABRIC and TCGA. Eosinophils were lower in both METABRIC and our cohorts. Monocytes were higher in TCGA and lower in our cohort. B cells were higher and plasma cells were lower in METABRIC. Activated MAST cells were lower and neutrophils were higher in our cohort. There was no correlation between tumor tissue and blood in lymphocyte, neutrophil, basophil, and monocyte counts (**Figure S8**). These results indicate that patients with high estrogen reactivity had low CYT and low levels of immunostimulatory cells in BC TME.

### Stimulatory and Inhibitory Factors of the Cancer-Immunity Cycle Were Decreased in High Estrogen-Reactive BC

**Figure 4:**
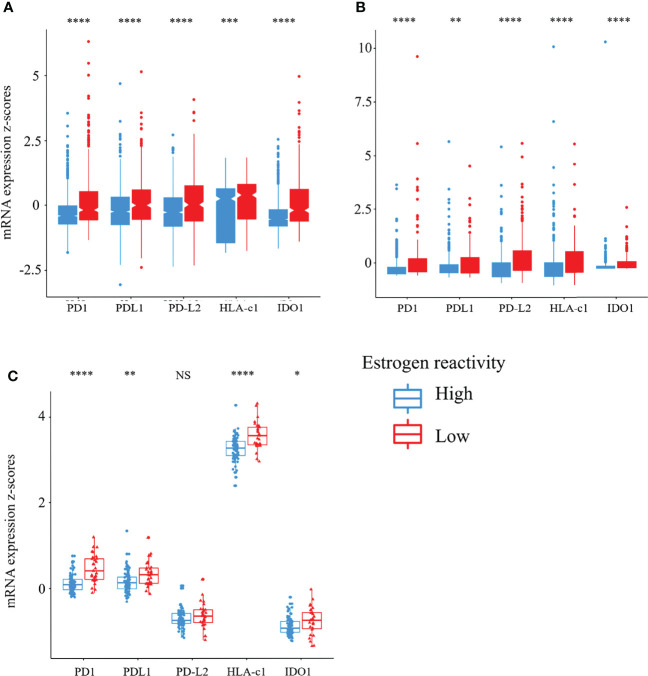
Stimulatory and inhibitory factors of the CIC in the levels of estrogen reactivity. Box plots of the relationship between estrogen reactivity and stimulatory (HLA-A mRNA) or inhibitory factors (PD-1, PD-L1, PD-L2, and IDO1) of the CIC in METABRIC **(A)** and TCGA **(B)** and our cohort **(C)** were shown. **** means P < 0.0001, *** means P < 0.001, ** means P < 0.01, and * means P < 0.05. CIC, cancer immunity cycle; METABRIC, Molecular Taxonomy of Breast Cancer International Consortium; TCGA, The Cancer Genome Atlas; NS, not significant.

The cancer-immunity cycle (CIC) is a series of self-sustaining stepwise events required to gain efficient control of cancer growth by the immune system (29). We reported that disability of CIC had a significant effect on TME and prognosis of BC patients (18). Here, we explored the relationship between the levels of estrogen reactivity and stimulatory or inhibitory factors of the CIC in all cohorts (**Figure 4**). In the analysis of stimulatory factors of cancer cells in the CIC, HLA-class I expression was lower in the high estrogen reactivity group in all cohorts. In the analysis of inhibitory factors of cancer cells in the CIC, the expressions of PD1, PD-L1, PD-L2, and IDO1 were lower in the high estrogen reactivity group in all cohorts. These results indicate that stimulatory and inhibitory factors of cancer cells were statistically significantly decreased in the high estrogen reactivity group.

### Patients With High Estrogen Reactivity Were Associated With Better RFS in All Cohorts

**Figure 5:**
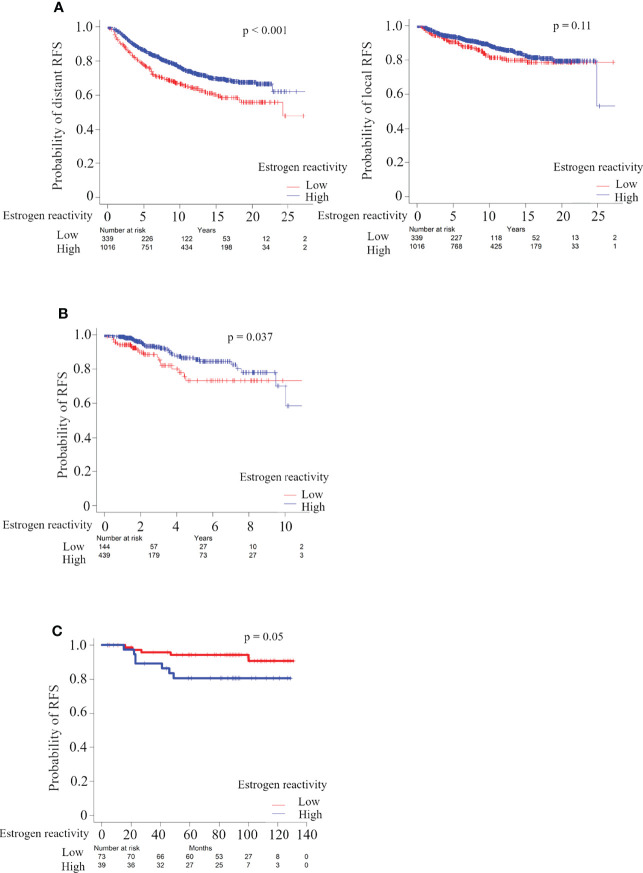
Analysis of the relationship between estrogen reactivity and survival in METABRIC and TCGA and our cohort. Kaplan-Meier plots of the association of estrogen reactivity with distant RFS in METABRIC **(A)** and RFS in TCGA **(B)** and in our cohort **(C)**. RES, recurrence-free survival; METABRIC, Molecular Taxonomy of Breast Cancer International Consortium; TCGA, The Cancer Genome Atlas; RFS, recurrent-free survival.

In order to investigate whether estrogen reactivity can serve as a prognostic biomarker, we examined the relationship between the levels of estrogen reactivity and prognosis, which were tested by the Kaplan–Meier method and verified by the log-rank test (**Figure 5A**). In the METABRIC HR+HER2- cohort, distant recurrence analysis showed that patients with high estrogen reactivity were significantly associated with better prognosis (**Figure 5A**, P < 0.0001). However, there were no significant differences between the levels of estrogen reactivity and local recurrence (P = 0.11). Patients with high estrogen reactivity were significantly associated with better RFS in TCGA HR+HER2- cohort (P = 0.037) and our cohort (P = 0.05). Despite differences in mortality rates between three cohorts, high estrogen reactivity was associated with better prognosis in the HR+ subtype.

## Discussion

Estrogen signals play an important role in the molecular and genomic pathways of the HR+ BC phenotype by affecting the transcriptional modulation of various genes such as proliferation regulators, growth factors, cell cycle, and apoptosis modulators (5, 6). The physical behavior of stromal cells, extracellular matrix, immune cells, macrophages, and soluble factors can modulate tumor cells and the TME to promote cancer survival or progression (8). Therefore, it was of interest to investigate estrogen reactivity in the BC microenvironment. We aimed to test the hypothesis that estrogen reactivity affects immune cells in the TME and gene expression profiles, and survival using GSVA scores of ESTROGEN_RESPONSE_EARLY and ESTROGEN_RESPONSE_LATE on collected data from METABRIC and TCGA BC cohorts and our institutional cohort.

This study generated three interesting results with clinical implications. First, high estrogen reactivity was related to Myc targets, metabolism-related gene sets, cell stress-responsive gene sets, TGF-beta signaling, androgen response, and MTORC1 signaling. Low estrogen reactivity was related to immune-related proteins, IL2-STAT5 signaling, IL6-JAK-STAT3 signaling, KRAS signaling, cell cycle-related gene sets, and EMT (**Figure 2**). No common pathway associated with the degree of estrogen reactivity in the three cohorts could be found except for estrogen response gene sets. This was a result of stricter cutoff criteria to identify pathways of comparable quality to estrogen reactivity, and the extracted pathways were considered significant, if not common, among the cohorts. Some pathways that have been found to be related to the degree of estrogen sensitivity have been reported as follows: Oxidative phosphorylation is regulated *via* ERβ in TNBC (30), and the adipogenesis pathway induces higher estrogen-reactive TME, thus enabling mammary tumorigenesis (31). Unfolded protein response signaling promotes a malignant phenotype in BC and can confer tumors with resistance to widely used therapies (32). There is a direct link between mTORC1 and ERα, which further implicates mTORC1 signaling in the pathogenesis of ER-positive BC (33). In the cell cycle, although the molecular mechanism underlying estrogenic regulation of the G1 phase is unclear, current evidence suggests that estrogens may regulate some important molecules required for transition to the S phase (34). Decreased estrogen signaling has been reported to result in potent EMT characterized by significant changes in the expression profile of certain matrix macromolecules (35).

Second, BC with high levels of estrogen reactivity has low immune cytolytic activity and low levels of immunostimulatory cells (**Figures 3** and **S5-7**). It also has low levels of stimulatory and inhibitory factors of the CIC (**Figure 4**). In GSEA, we confirmed that the degree of estrogen reactivity was related to immune-related proteins (**Figure 2**). It has been reported that estrogen signals have been implicated in several effects of immunity and autoimmunity (36, 37). These studies further showed that estrogen signals affect the size, maturation, and development of T cells. In CD4+ T cells, estrogen signals are associated with the inhibition of the production of Th1 pro-inflammatory cytokines and the promotion of Th2 anti-inflammatory cytokines. E2 through the ERα pathway also impacts the activation and survival of B cells. Aside from lymphocytes, estrogen exposure delays apoptosis in human neutrophils, causes a reduction in NK cell cytotoxic activity, and modulates different macrophage actions and their metabolism. We showed that low expressions of estrogen-responsive genes were correlated with the presence of lymphocytes with cytolytic activity (18). In summary, lymphocytes, fibroblasts, neutrophils, eosinophils, macrophages, and myeloid-derived suppressor cells present in the TME are disturbed by estrogen signaling, which affects the growth and migration of cancer cells. Further, it has been revealed that the immune response in tumor tissue reflects a series of carefully regulated events that can be optimally addressed as a group rather than individual cells (29). CIC is defined as a series of self-sustaining stepwise events required to gain efficient control of cancer growth by the immune system (29), and the association between CIC and estrogen signaling remains unknown. We showed that high estrogen sensitivity was correlated with CIC dysfunction (18). These results demonstrate that high estrogen reactivity not only reduces the distribution of antitumor immune cells in the TME but also inactivates the antitumor immune system.

Finally, patients with high estrogen reactivity were associated with a better prognosis. We showed that, despite differences in mortality rates between three cohorts, high estrogen reactivity was associated with better prognosis in the HR+ subtype (**Figure 5**). It has become clear that sets of genes that are associated with increased risk of metastasis mostly represent proliferation, estrogen receptor-associated genes, and immune cell-associated genes (6). We reported that ESTROGEN_RESPONSE_EARLY score was associated with survival in primary and metastatic BC (38). In this study, we proved that the levels of estrogen reactivity, which was calculated more precisely by combining ESTROGEN_RESPONSE_EARLY score and ESTROGEN_RESPONSE_LATE score, was related to the levels of immune cells as described above, and further to distant RFS (**Figures 5** and **S9**). These results demonstrate that high estrogen reactivity is a prognostic factor and can be a means of predicting distant metastases.

Although the study demonstrates promising results, it has limitations. Although we utilized multiple large patient cohorts, this is a retrospective study utilizing publicly available datasets and our own institutional data. Second, each cohort we surveyed has different patient backgrounds and clinical characteristics. In METABRIC and TCGA, some of the clinical factors deemed necessary were missing. TCGA cohort is known to lack comorbidities and therapeutic intervention data. Our institutional cohort has sufficient clinical data but a smaller number of patients. Differences between cohorts were noticeable in “Association of estrogen reactivity with clinical features” and “Gene expression profiles by the levels of estrogen reactivity.” Therefore, it is necessary to verify the obtained with other large cohorts with sufficient clinical data. Third, we could not investigate the association of the estrogen reactivity with the effects of drugs used in recurrent BC, especially CDK4/6 inhibitors. Finally, this study does not include *in vitro* or *in vivo* experiments. Therefore, the mechanism for further understanding the results has not been clarified. For example, it is not clear whether the observed estrogen reactivity is of tumor origin or immune cell origin. It is not clear whether immune infiltration in TME is directly regulated by E2 or indirectly by the E2 response of tumor cells.

## Conclusions

We demonstrated the relationship between estrogen reactivity and immune cells and gene expression profiles and survival. We revealed that estrogen reactivity would perturb multiple pathways and high estrogen reactivity would act suppressively against the immune microenvironment and act as a prognostic factor. Based on these reported results, we anticipate that further research can be conducted to establish a greater understanding of the role of estrogen reactivity to TME of BC.

## Data Availability Statement

The original contributions presented in the study are included in the article/**Supplementary Material**. Expression profiles are available in the online data repository Gene Expression Omnibus database (GSE199135). Further inquiries can be directed to the corresponding author.

## Ethics Statement

The studies involving human participants were reviewed and approved by the Ethics Committee of Hokkaido University Hospital (protocol code: 14-046, October 3, 2014, Hokkaido, Japan). The patients/participants provided their written informed consent to participate in this study.

## Author Contributions

TT conceptualized and performed the analysis and prepared the article. TT, YT, MO, RW and WT performed the analysis. YH and KH provided the data. TT provided the supervision and prepared the article. LY performed the analysis and supervised the analysis. AP and KT prepared the article. All authors contributed to the article and approved the submitted version.

## Funding

This work was supported by an NIH grant R01CA160688 to KT, and an NCI grant P30CA016056 involving the use of Roswell Park Cancer Institute’s Bioinformatics and Biostatistics Shared Resources.

## Conflict of Interest

The authors declare that the research was conducted in the absence of any commercial or financial relationships that could be construed as a potential conflict of interest.

## Publisher’s Note

All claims expressed in this article are solely those of the authors and do not necessarily represent those of their affiliated organizations, or those of the publisher, the editors and the reviewers. Any product that may be evaluated in this article, or claim that may be made by its manufacturer, is not guaranteed or endorsed by the publisher.

## Abbreviations

BC, breast cancer; ER+, estrogen receptor positive; E2, estradiol; TME, tumor microenvironment; GSVA; Gene Set Variation Analysis; METABRIC, Molecular Taxonomy of Breast Cancer International Consortium; TCGA, The Cancer Genome Atlas; FFPE, formalin-fixed paraffin-embedded; PgR, progesterone receptor; GSEA, gene set enrichment analyses; NES, normalized enrichment score; FDR, false discovery rate; CYT, immune cytolytic activity; RFS, recurrence-free survival; HR, hormone receptor; HER2, human epidermal growth factor receptor 2; TN, triple negative; IFN, interferon; EMT, epithelial-mesenchymal transition; Tregs, regulatory T cells; CIC, cancer-immunity cycle.
