# Supplementary material for: Clinical Relevance of Estrogen Reactivity in the Breast Cancer Microenvironment
Source: Front Oncol. 2022 May 23;12:865024. doi: 10.3389/fonc.2022.865024 (PMC9169154; doi:10.3389/fonc.2022.865024)

**Supplementary files**

**Table S1:** Patients and clinical characteristics associated with estrogen reactivity in METABRIC and TCGA cohort

| Variables | | Number of Patients (%) | | | | | | | |
| --- | --- | --- | --- | --- | --- | --- | --- | --- | --- |
|  |  | METABRIC | | | | TCGA | | | |
|  |  | Total | Estrogen reactivity | | | Total | Estrogen reactivity | | |
|  |  |  | High | Low | *P*-value |  | High | Low | *P*-value |
|  |  | *N* = 1355 | *N* = 1016 | *N* = 339 |  | *N* = 585 | *N* = 440 | *N* = 145 |  |
| Age | 50 > | 220 (16.2) | 183 (18) | 37 (10.9) | 0.002* | 147 (25.1) | 120 (27.3) | 27 (18.6) | 0.037* |
|  | 50 < | 1135 (83.8) | 833 (82) | 302 (89.1) |  | 438 (74.9) | 320 (72.7) | 118 (81.4) |  |
| Menopausal state | Pre | 220 (16.2) | 183 (18) | 37 (10.9) | 0.002* | 134 (22.9) | 112 (25.5) | 22 (15.2) | 0.0075* |
|  | Post | 1135 (83.8) | 833 (82) | 302 (89.1) |  | 389 (66.5) | 280 (63.6) | 109 (75.2) |  |
|  | Unknown | 0 | 0 | 0 |  | 62 (10.6) | 48 (10.9) | 14 (9.7) |  |
| Tumor size (cm) | 2 > | 601 (44.4) | 456 (44.9) | 145 (42.8) | 0.5 | 100 (17.1) | 371 (84.3) | 114 (78.6) | 0.11 |
|  | 2 < | 742 (54.8) | 551 (54.2) | 191 (56.3) |  | 485 (82.9) | 69 (15.7) | 31 (21.4) |  |
|  | Unknown | 12 (0.9) | 9 (0.9) | 3 (0.9) |  | 0 | 0 | 0 |  |
| Lymphnode | Negative | 745 (55) | 573 (56.4) | 172 (50.7) | 0.07 | 269 (46) | 198 (45) | 71 (49) | 0.4 |
|  | Positive | 610 (45) | 443 (43.6) | 167 (49.3) |  | 308 (52.6) | 236 (53.6) | 72 (49.7) |  |
| Histopathology | Ductal | 979 (72.3) | 732 (72) | 247 (72.9) | 0.88 | 385 (65.8) | 299 (68) | 86 (59.3) | 0.14 |
|  | Lobular | 118 (8.7) | 89 (8.8) | 29 (8.6) |  | 144 (24.6) | 100 (22.7) | 44 (30.3) |  |
|  | Others/ unknown | 248 (18.3) | 189 (18.6) | 59 (17.4) |  | 56 (9.6) | 41 (9.3) | 15 (10.3) |  |
| Tumor grade | 1 | 159 (11.7) | 127 (12.5) | 32 (9.4) | 0.12 |  |  |  |  |
|  | 2,3 | 1135 (83.8) | 842 (82.9) | 293 (86.4) |  |  |  |  |  |
|  | unknown | 61 (4.5) | 47 (4.6) | 14 (4.1) |  |  |  |  |  |
| Clinical Stage | I/II | 931 (68.7) | 702 (69.1) | 229 (67.6) | 0.12 | 433 (74) | 327 (74.3) | 106 (73.1) | 0.77 |
|  | III/IV | 70 (5.2) | 47 (4.6) | 23 (6.8) |  | 144 (24.6) | 107 (24.3) | 37 (25.5) |  |
|  | Unknown | 354 (26.1) | 267 (26.3) | 87 (25.7) |  | 0 | 0 | 0 |  |
| PgR | Negative | 411 (30.3) | 225 (22.1) | 186 (54.9) | <0.0001* | 80 (13.7) | 40 (9.1) | 40 (27.6) | <0.0001* |
|  | Positive | 944 (69.7) | 791 (77.9) | 153 (45.1) |  | 503 (86) | 398 (90.5) | 105 (72.4) |  |
|  | Unknown | 0 | 0 | 0 |  | 2 (0.3) | 2 (0.5) | 0 |  |
| Molecular Characterization | Luminal A | 656 (48.4) | 554 (54.5) | 102 (30.1) | <0.0001* | 303 (51.8) | 252 (57.3) | 51 (35.2) | NS |
|  | Luminal B | 419 (30.9) | 313 (30.8) | 106 (31.3) |  | 122 (20.9) | 91 (20.7) | 31 (21.4) |  |
|  | HER2 | 63 (4.6) | 29 (2.9) | 34 (10) |  | 3 (0.5) | 0 | 3 (2.1) |  |
|  | Basal-like | 25 (1.8) | 6 (0.6) | 19 (5.6) |  | 9 (1.5) | 1 (0.2) | 8 (5.5) |  |
|  | Claudin-low | 72 (5.3) | 21 (2.1) | 51 (15) |  | 13 (2.2) | 5 (1.1) | 8 (5.5) |  |
|  | Normal | 114 (8.4) | 89 (8.8) | 25 (7.4) |  | 135 (23.1) | 91 (20.7) | 44 (30.3) |  |
| Adjuvant Endocrine therapy | No | 389 (28.7) | 307 (30.2) | 82 (24.2) | 0.034* |  |  |  |  |
|  | Yes | 966 (71.3) | 709 (69.8) | 257 (75.8) |  |  |  |  |  |
| Adjuvant chemotherapy | No | 1225 (90.4) | 930 (91.5) | 295 (87) | 0.015* |  |  |  |  |
|  | Yes | 130 (9.6) | 86 (8.5) | 44 (13) |  |  |  |  |  |

**Abbreviations**: METABRIC, Molecular Taxonomy of Breast Cancer International Consortium; TCGA, The Cancer Genome Atlas; PgR, progesterone receptor.

* Factor showing statistical significance. The chi-square test and Fisher’s extract test were used to assess baseline differences between binary variables. P < .05 is considered statistically significant

**Table S2:** Patients and clinical characteristics associated with estrogen reactivity in our cohort.

| Variables | | Number of Patients (%) | | | |
| --- | --- | --- | --- | --- | --- |
|  |  | Total | Estrogen reactivity | |  |
|  |  |  | High | Low | *P*-value |
|  |  | (*N* = 112) | (*N* = 73) | (*N* = 39) |  |
| Age | 50 > | 28 (25) | 20 (27.4) | 8 (20.5) | 0.42 |
|  | 50 < | 84 (75) | 53 (72.6) | 31 (79.5) |  |
| Menopausal state | Pre | 34 (30.4) | 24 (32.9) | 10 (25.6) | 0.43 |
|  | Post | 78 (69.6) | 49 (67.1) | 29 (74.4) |  |
| Tumor size (cm) | 2 > | 79 (70.5) | 54 (74) | 25 (64.1) | 0.48 |
|  | 2 < | 31 (27.7) | 19 (26) | 12 (30.8) |  |
|  | Unknown | 2 (1.8) | 0 | 2 (5.1) |  |
| Lymphnode | Negative | 71 (63.4) | 48 (65.8) | 23 (59) | 0.48 |
|  | Positive | 41 (36.6) | 25 (34.2) | 16 (41) |  |
| Histopathology | Ductal | 110 (98.2) | 72 (98.6) | 38 (97.4) | NA |
|  | Lobular | 1 (0.9) | 1 (1.4) | 0 |  |
|  | Others | 1 (0.9) | 0 | 1 (2.6) |  |
| Tumor grade | 1 | 37 (33) | 28 (38.3) | 9 (23.1) | 0.13 |
|  | 2,3 | 72 (64.3) | 44 (60.3) | 28 (71.8) |  |
|  | unknown | 3 (2.7) | 1 (1.4) | 2 (5.1) |  |
| Clinical stage | I/II | 109 (97.3) | 73 (100) | 36 (92.3) | NA |
|  | III | 2 (1.8) | 0 | 2 (5.1) |  |
|  | unknown | 1 (0.9) | 0 | 1 (2.6) |  |
| HER2 | Negative | 101 (90.2) | 66 (90.4) | 35 (89.7) | 0.87 |
|  | Positive | 8 (7.1) | 5 (6.8) | 3 (7.7) |  |
|  | Unknown | 3 (2.7) | 2 (2.8) | 1 (2.6) |  |
| Ki67 | 20 % > | 78 (69.6) | 54 (74) | 24 (61.5) | 0.15 |
|  | 20 % < | 26 (23.2) | 14 (19.1) | 12 (30.8) |  |
|  | Unknown | 8 (7.1) | 5 (6.9) | 3 (7.7) |  |
| Adjuvant Endocrine therapy | No | 3 (2.7) | 2 (2.8) | 1 (2.6) | 0.97 |
|  | Yes | 108 (96.4) | 71 (97.2) | 37 (94.9) |  |
| Adjuvant chemotherapy | No | 89 (79.5) | 60 (82.2) | 29 (74.4) | 0.46 |
|  | Yes | 22 (19.6) | 13 (17.8) | 9 (23.1) |  |

**Abbreviations**: HER2, human epidermal growth factor receptor 2.

**Table S3: List of pre-ranked GSEA with high/low estrogen reactivity in METABRIC cohort.**

| **HALLMARK_NAME** | NES | FDR q-val |
| --- | --- | --- |
| **ESTROGEN_RESPONSE_EARLY** | 3.69 | 0.0001 |
| **ESTROGEN_RESPONSE_LATE** | 3.21 | 0.005 |
| **IL6_JAK_STAT3_SIGNALING** | -1.97 | 0.0001 |
| **G2M_CHECKPOINT** | -1.98 | 0.0001 |
| **KRAS_SIGNALING_UP** | -2.06 | 0.0001 |
| **E2F_TARGETS** | -2.14 | 0.0001 |
| **IL2_STAT5_SIGNALING** | -2.17 | 0.0001 |
| **COMPLEMENT** | -2.2 | 0.0001 |
| **INFLAMMATORY_RESPONSE** | -2.32 | 0.0001 |
| **INTERFERON_ALPHA_RESPONSE** | -2.53 | 0.0001 |
| **INTERFERON_GAMMA_RESPONSE** | -2.95 | 0.0001 |
| **ALLOGRAFT_REJECTION** | -3.25 | 0.0001 |

**Abbreviations**: GSEA, gene set enrichment analysis; METABRIC, Molecular Taxonomy of Breast Cancer International Consortium; NES, normalized enrichment score; FDR, false discovery rate.

Gene sets that met a threshold of NES >1.8 or <-1.8 and FDR q-value < 0.01 were significantly enriched.

**Table S4: List of pre-ranked GSEA with high/low estrogen reactivity in TCGA cohort.**

| **HALLMARK_NAME** | NES | FDR q-val |
| --- | --- | --- |
| **ESTROGEN_RESPONSE_EARLY** | 4.13 | 0.0001 |
| **ESTROGEN_RESPONSE_LATE** | 3.47 | 0.005 |
| **EPITHELIAL_MESENCHYMAL_TRANSITION** | -1.85 | 0.0001 |
| **IL2_STAT5_SIGNALING** | -1.87 | 0.0001 |
| **COMPLEMENT** | -1.88 | 0.0001 |
| **IL6_JAK_STAT3_SIGNALING** | -1.94 | 0.0001 |
| **KRAS_SIGNALING_UP** | -1.96 | 0.0001 |
| **INTERFERON_GAMMA_RESPONSE** | -2.14 | 0.0001 |
| **INFLAMMATORY_RESPONSE** | -2.15 | 0.0001 |
| **ALLOGRAFT_REJECTION** | -2.48 | 0.0001 |

**Abbreviations**: GSEA, gene set enrichment analysis; TCGA, The Cancer Genome Atlas; NES, normalized enrichment score; FDR, false discovery rate.

Gene sets that met a threshold of NES >1.8 or <-1.8 and FDR q-value < 0.01 were significantly enriched.

**Table S5: List of pre-ranked GSEA with high/low estrogen reactivity in our cohort.**

| **HALLMARK_NAME** | NES | FDR q-val |
| --- | --- | --- |
| **MYC_TARGETS_V1** | 3.21 | 0.0001 |
| **PROTEIN_SECRETION** | 2.98 | 0.0001 |
| **ESTROGEN_RESPONSE_EARLY** | 2.6 | 0.0001 |
| **UNFOLDED_PROTEIN_RESPONSE** | 2.46 | 0.0001 |
| **TGF_BETA_SIGNALING** | 2.31 | 0.0001 |
| **OXIDATIVE_PHOSPHORYLATION** | 2.27 | 0.0001 |
| **ANDROGEN_RESPONSE** | 2.21 | 0.0001 |
| **UV_RESPONSE_DN** | 2.16 | 0.0001 |
| **MTORC1_SIGNALING** | 1.96 | 0.004 |
| **ESTROGEN_RESPONSE_LATE** | 1.85 | 0.005 |
| **ADIPOGENESIS** | 1.83 | 4.00E-03 |
| **KRAS_SIGNALING_DN** | -1.84 | 1.00E-04 |

**Abbreviations**: GSEA, gene set enrichment analysis; NES, normalized enrichment score; FDR, false discovery rate.

Gene sets that met a threshold of NES >1.8 or <-1.8 and FDR q-value < 0.01 were significantly enriched.

**Figure S1.** The association of estrogen response scores in GSVA with breast cancer subtype. Boxplots of the comparison of estrogen response early and late score by subtype; HR+/ HER2-, HER2+, and TN were shown. **** means P<0.0001. **Abbreviations**: GSVA, Gene Set Variant Analysis; METABRIC, Molecular Taxonomy of Breast Cancer International Consortium; TCGA, The Cancer Genome Atlas; HR, hormone receptor; HER2, human epidermal growth receptor 2; TN, triple negative.

**
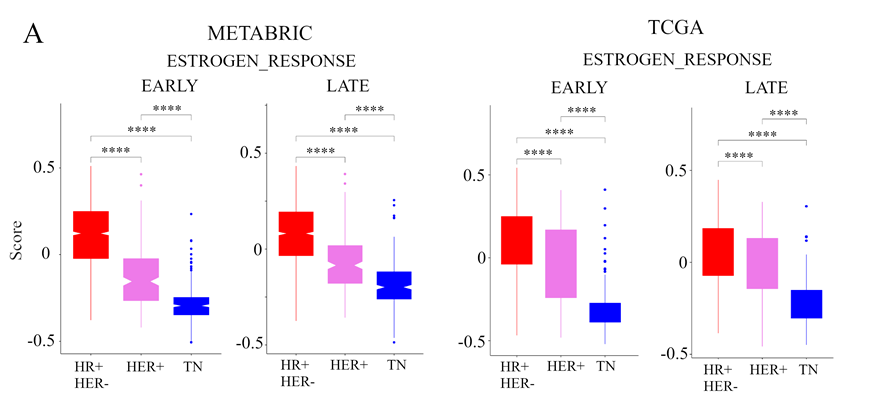
**

**Figure S2.** Correlation between ESTROGEN_RESPONSE_EARLY score and ESTROGEN_RESPONSE_LATE score by subtypes; whole, HR+/ HER2-, HER2+, and TN, in METABRIC and TCGA and our cohort. **Abbreviations**: HR, hormone receptor; HER2, human epidermal growth factor receptor 2; TN, triple negative; METABRIC, Molecular Taxonomy of Breast Cancer International Consortium; TCGA, The Cancer Genome Atlas; ERE, ESTROGEN_RESPONSE_EARLY score; ERL, ESTROGEN_RESPONSE_LATE　score.


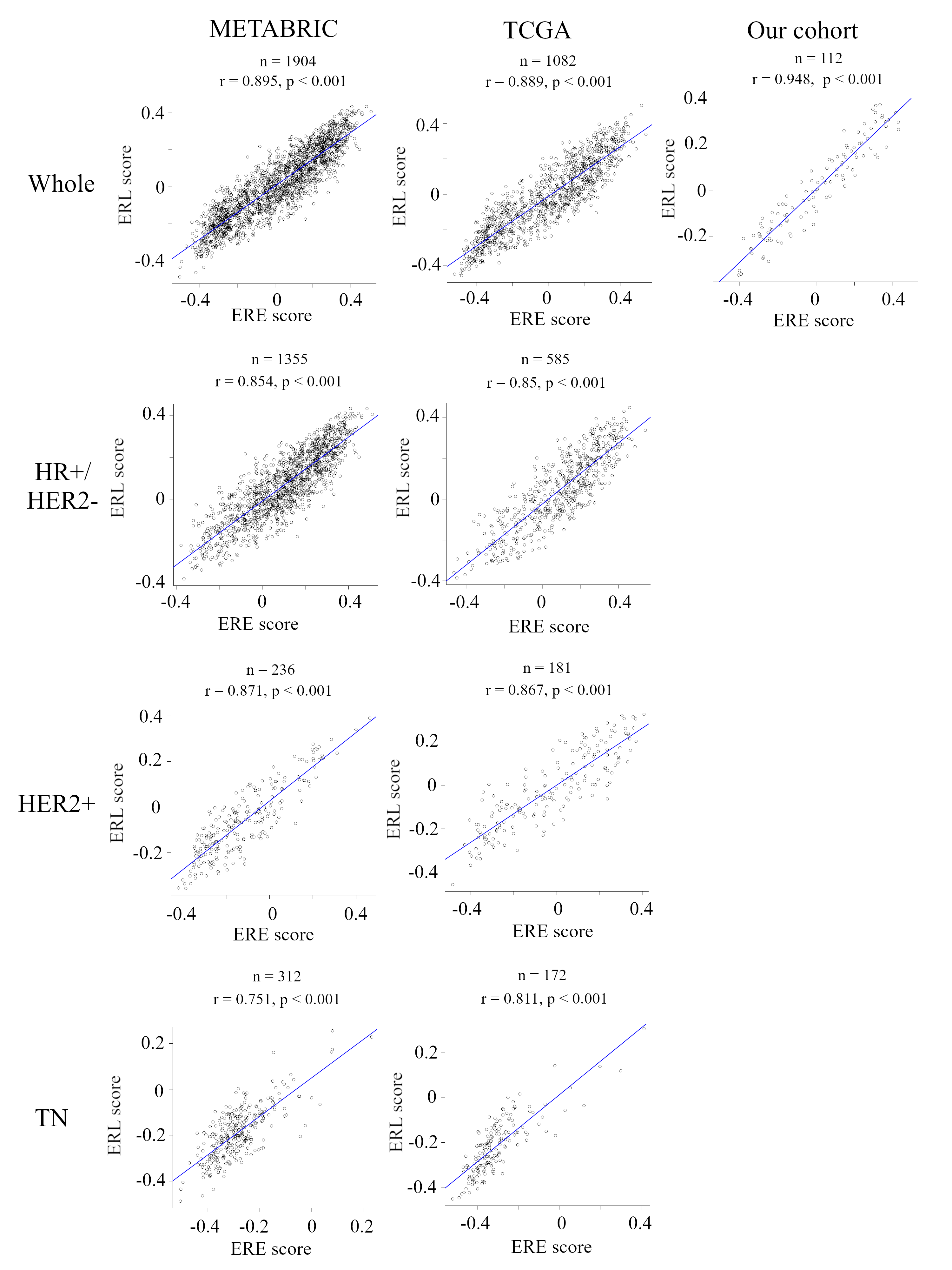


**Figure S3.** The association of estrogen response scores in GSVA with estrogen reactivity. Boxplots of the comparison of estrogen response early and late score by estrogen reactivity in whole cohort, (**A**), and in HER2+, (**B**), and TN, (**C**) in METABRIC and TCGA cohorts were shown. **** means P<0.0001. **Abbreviations**: GSVA, Gene Set Variant Analysis; HER2, human epidermal growth factor receptor2; TN, triple negative; METABRIC, Molecular Taxonomy of Breast Cancer International Consortium; TCGA, The Cancer Genome Atlas; HR, hormone receptor.


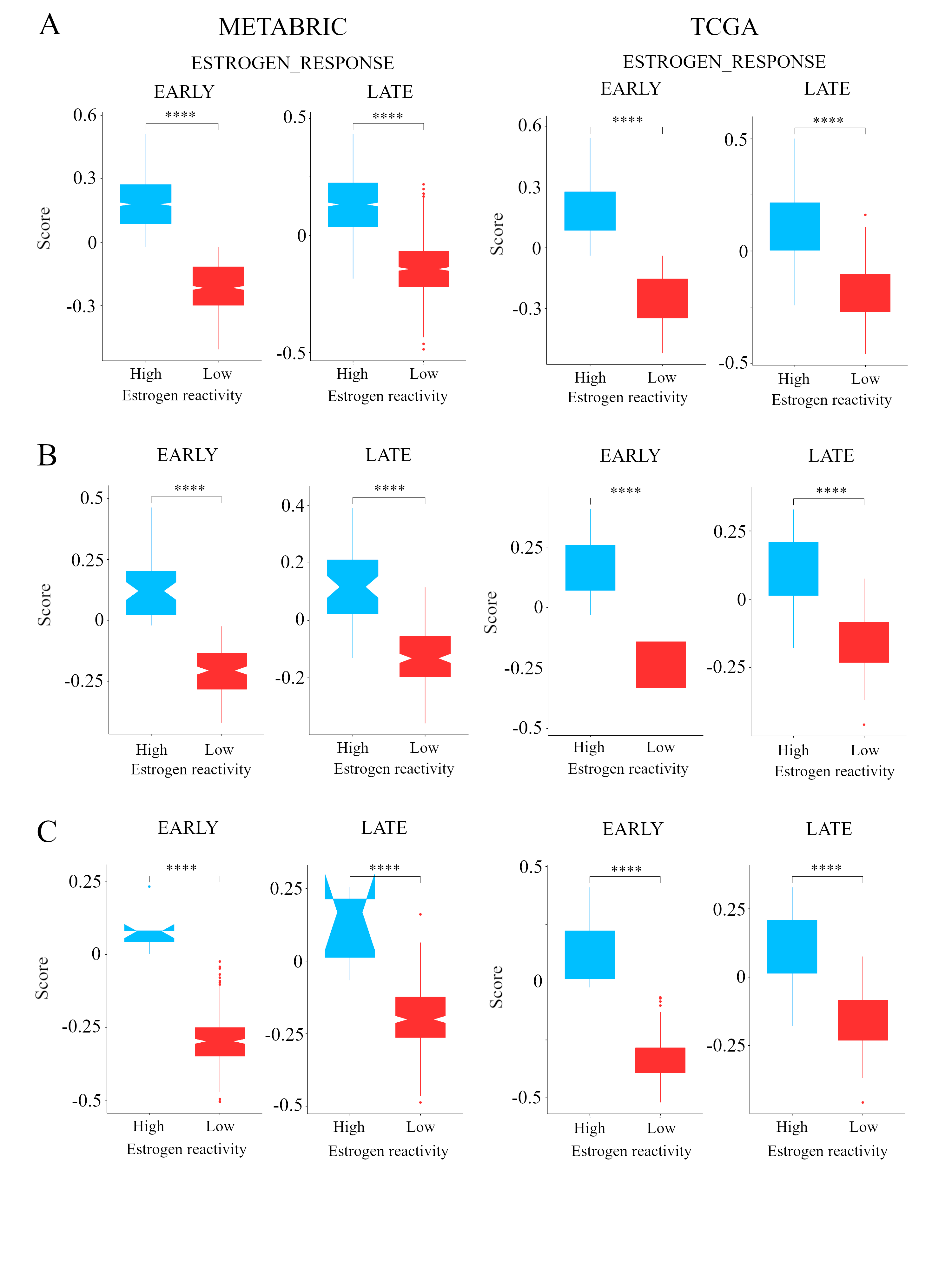


**Figure S4.** The association of estrogen reactivity and immunohistochemistry for ER and PgR. Boxplots of the comparison of ER and PgR percentage by estrogen reactivity in our cohort (**A**) and representative microscopic views of ER and PgR staining by high and low of estrogen reactivity (**B**) were shown. ** means P<0.01. **Abbreviations**: ER, estrogen receptor; PgR, progesterone receptor.


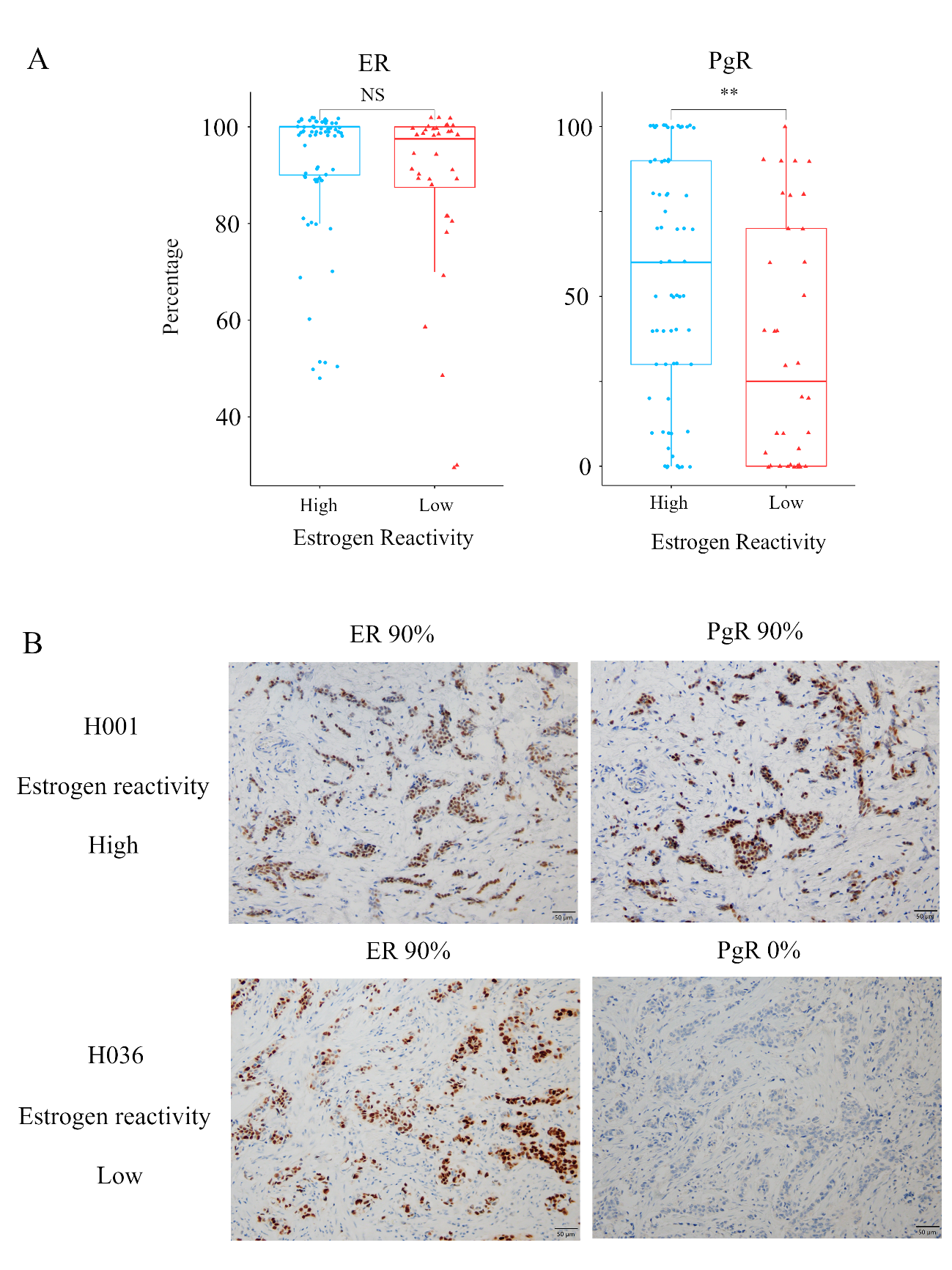


**Figure S5.** Verification of the relationship between estrogen reactivity and immune cell fractions using xCell in METABRIC. Box plots of the relationship between estrogen reactivity and immune cell fractions were shown. **** means P < 0.0001, *** means P < 0.001, ** means P<0.01 and * means P < 0.05. **Abbreviations**: METABRIC, Molecular Taxonomy of Breast Cancer International Consortium.

**
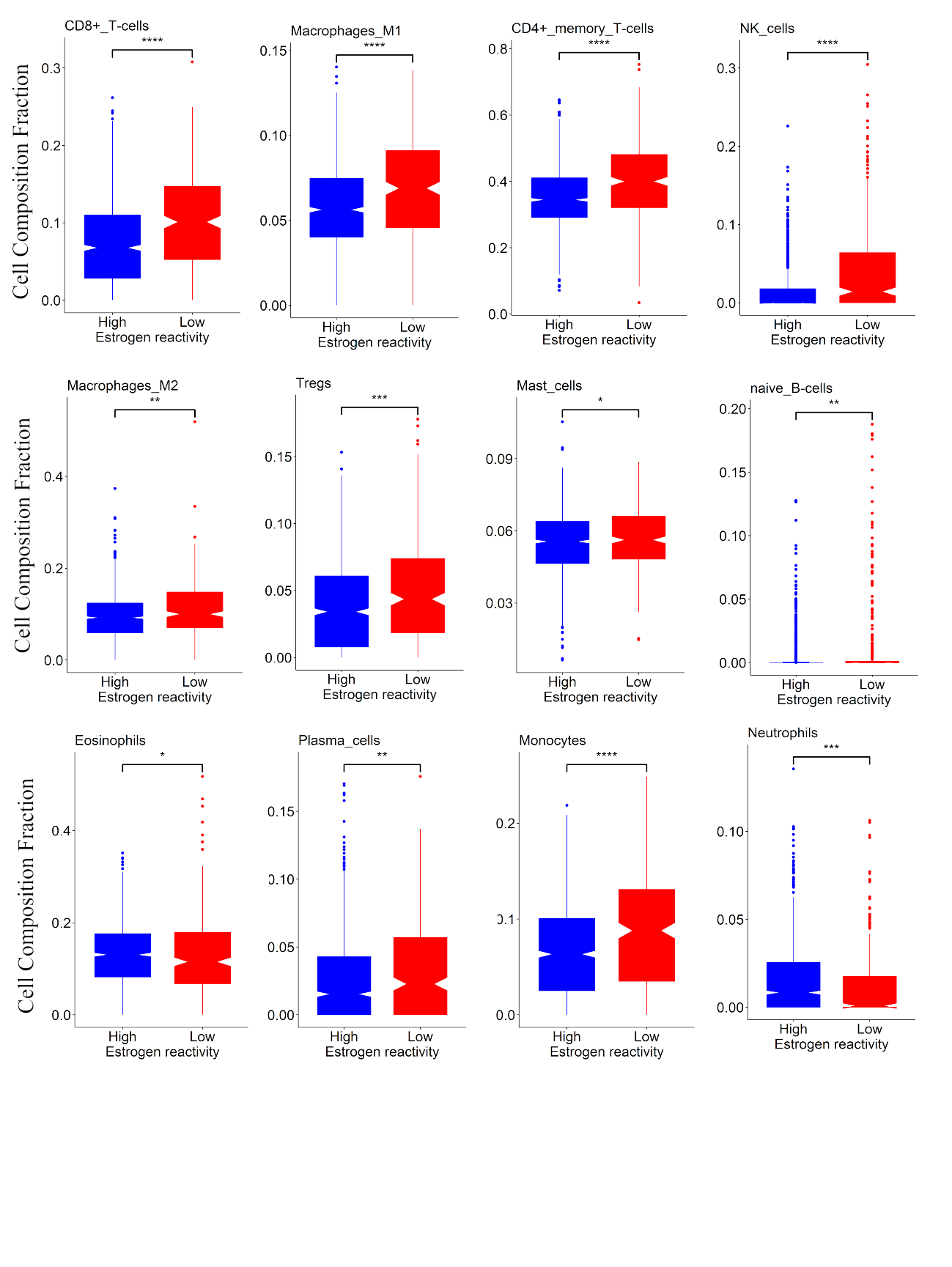
**

**Figure S6.** Verification of the relationship between estrogen reactivity and immune cell fractions using xCell in TCGA. Box plots of the relationship between estrogen reactivity and immune cell fractions were shown. **** means P < 0.0001, *** means P < 0.001, ** means P<0.01 and * means P < 0.05. **Abbreviations**: TCGA, The Cancer Genome Atlas, NS, not significant.


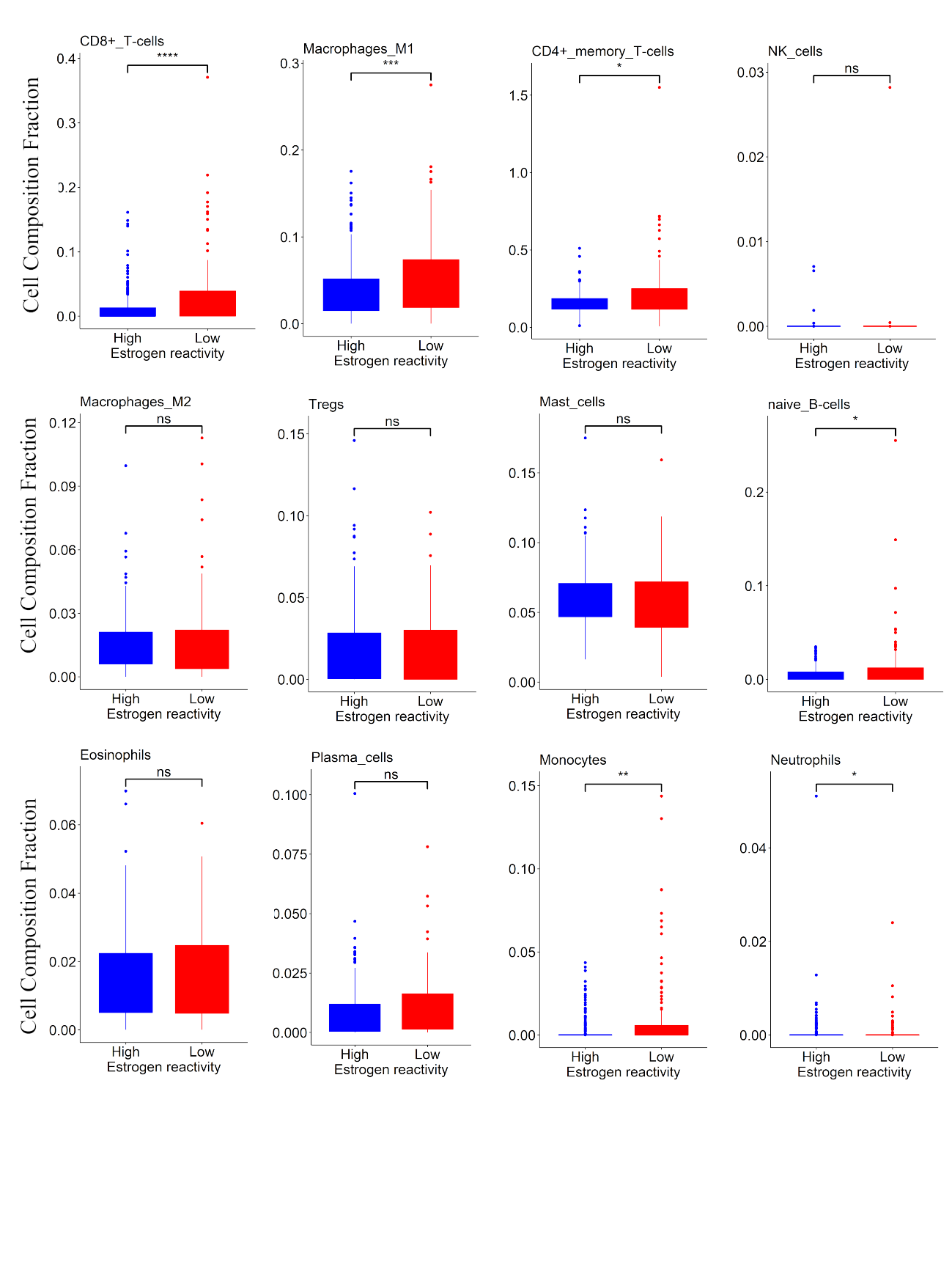


**Figure S7.** Verification of the relationship between estrogen reactivity and immune cell fractions using xCell in our institutional cohort. Box plots of the relationship between estrogen reactivity and immune cell fractions were shown. **** means P < 0.0001, *** means P < 0.001, ** means P<0.01 and * means P < 0.05. **Abbreviations**: NS, not significant.

**
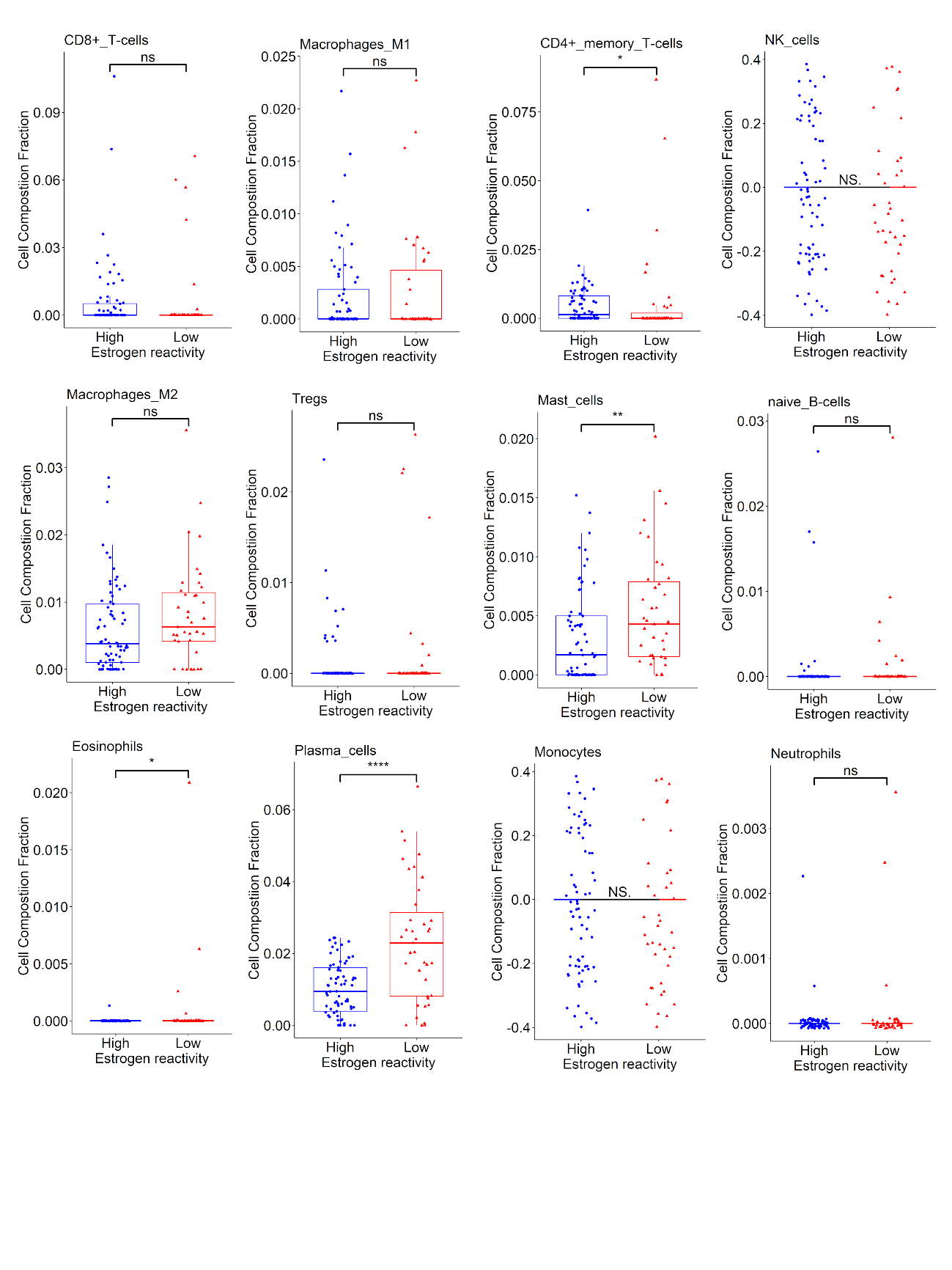
**

**Figure S8.** Correlation of the presence of neutrophils, basophils, monocytes, and lymphocytes between tumor tissue and blood in our cohort.


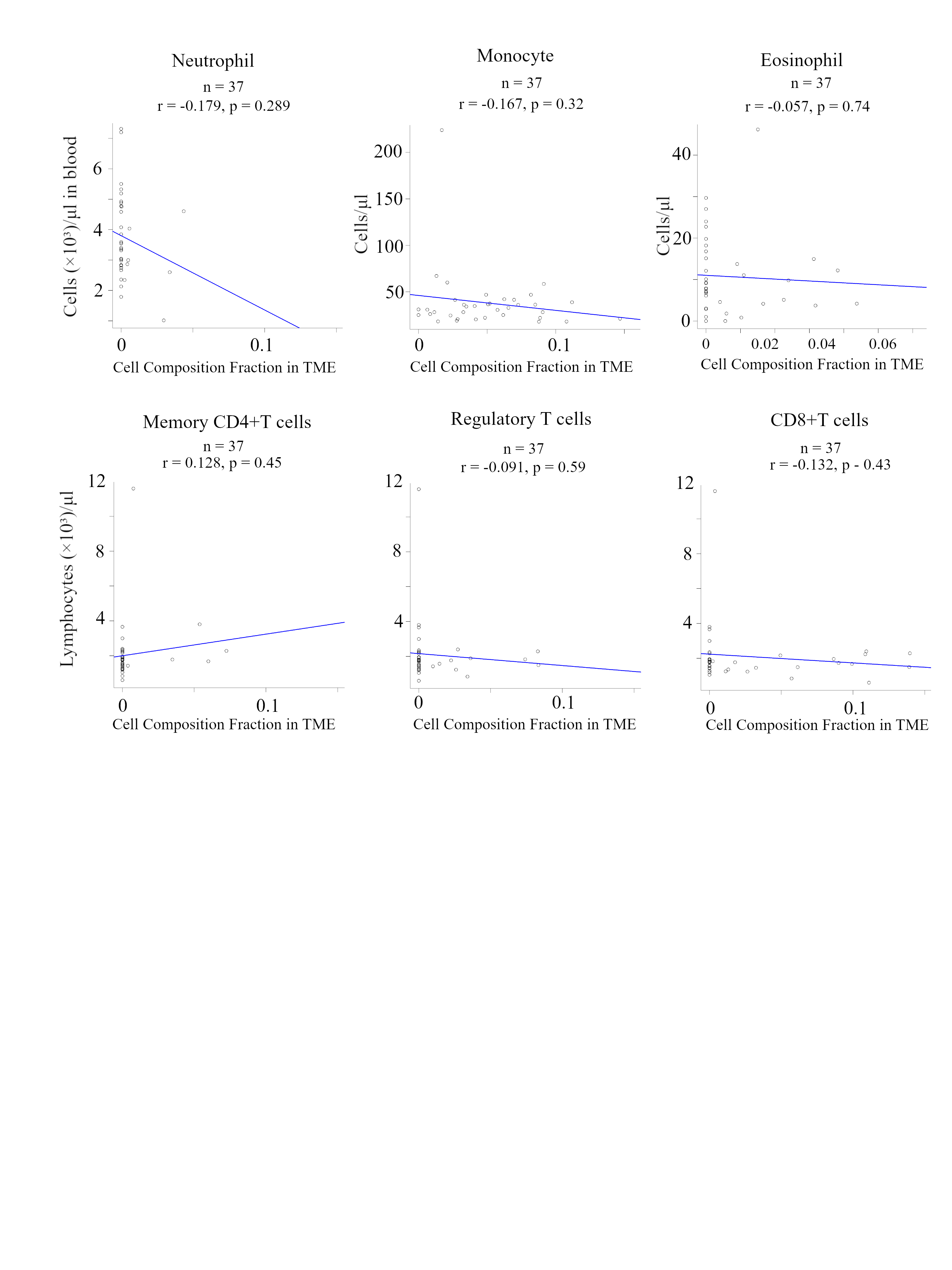


**Figure S9.** Analysis of the relationship between estrogen reactivity and survival by subtypes in METABRIC and TCGA cohort. Kaplan-Meier plots of the association of estrogen reactivity with distant RFS (**A**) and local RFS (**B**) in METABRIC and RFS in TCGA (**C**) by subtypes; (left to right) the whole cohort, the HER2+ group, and the TN group. **Abbreviations**: METABRIC, Molecular Taxonomy of Breast Cancer International Consortium; TCGA, The Cancer Genome Atlas; RFS, recurrent-free survival; RFS, recurrent-free survival; HER2+, human epidermal growth factor receptor2; TN, triple negative; HR, hormone receptor; Er, estrogen reactivity.


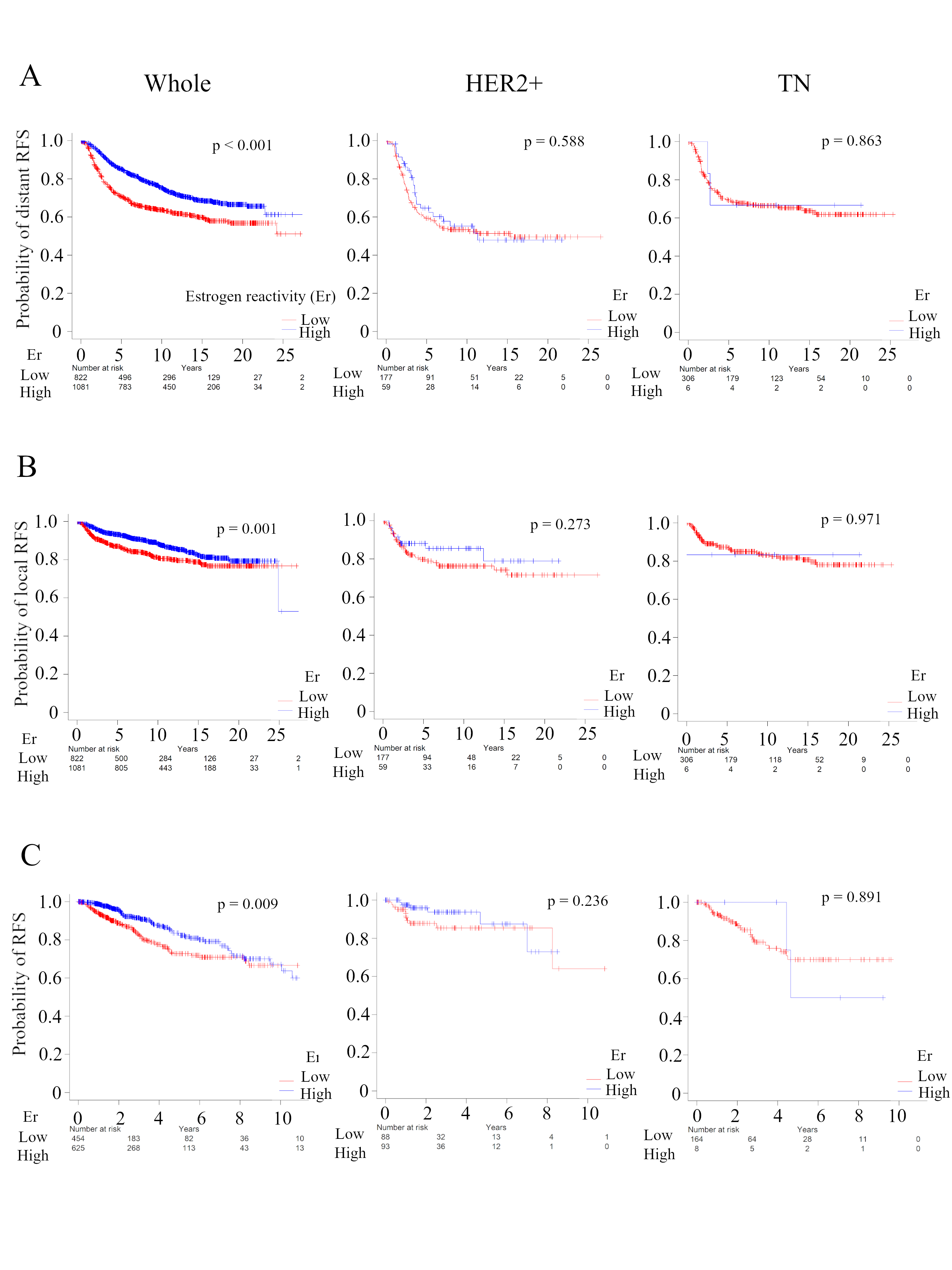

Supplement: Supplementary file 1 [file DataSheet_1.docx]
